# Supplementary material for: Knockdown of the snoRNA-Jouvence Blocks the Proliferation and Leads to the Death of Human Primary Glioblastoma Cells
Source: Noncoding RNA. 2025 Jul 18;11(4):54. doi: 10.3390/ncrna11040054 (PMC12286020; doi:10.3390/ncrna11040054)
Supplement: Supplementary file 1 [file ncrna-11-00054-s001.zip › ncrna-3676045-supplementary---2.pdf]

Knockdown of the snoRNA-jouvence blocks the cell proliferation and leads to cell death of human primary cancerous glioblastoma cells

Lola Jaque-Cabrera[1], Julia Buggiani[1#], Jérôme Bignon[2], Patricia Daira[3], Nathalie Bernoud-Hubac[3], and Jean-René Martin[1*]

Supplementary Figures

Supplementary Figure 1)

a) Vector map of the newly designed and synthetized sh-lentivirus anti-jouvence. The sh-anti-jouvence human snoRNA is placed under the U6-promoter. The vector also contains the markers: EGFP:T2A:Puromycine. The target sequence is also indicated.

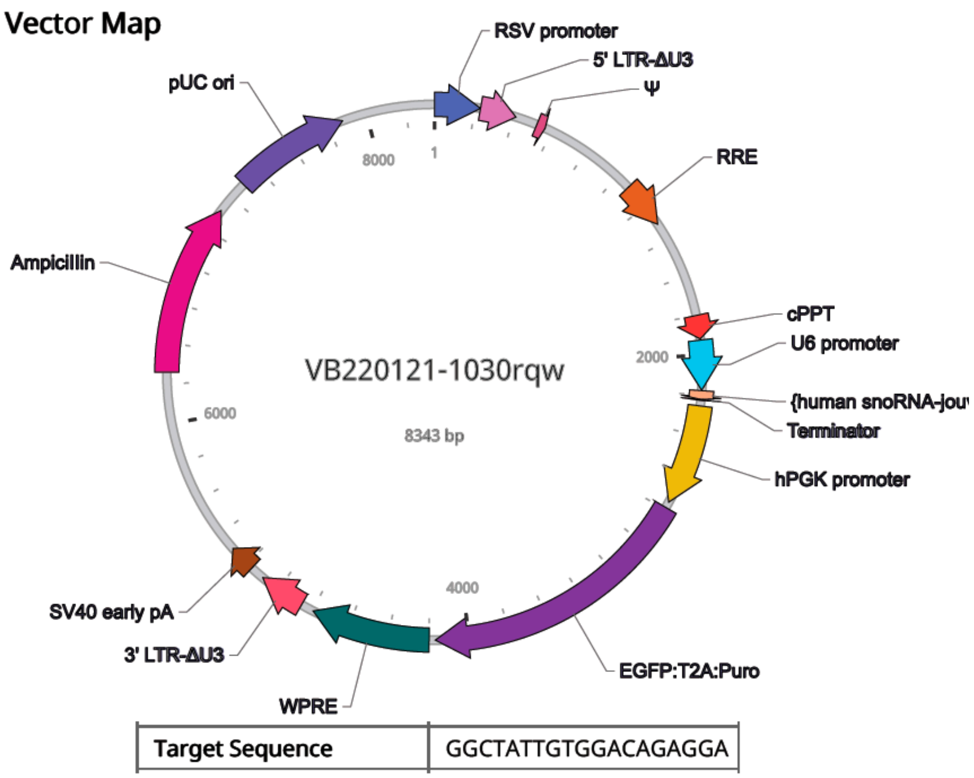

b) Dose response effect of different MOI of the sh-lentivirus transduction. (NT= non-transduced, Pb = polybrene, sh-co = sh-scramble-lentivirus at MOI-20, others = sh-RNA-anti-jouvence at different MOI).

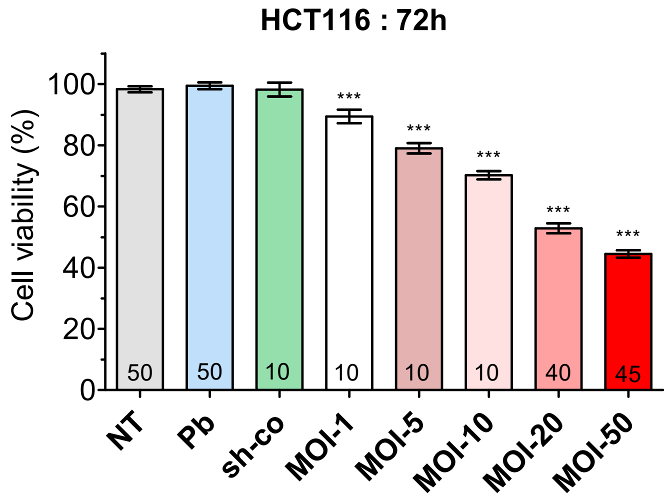

Supplementary Figure 2)

Efficiency of the sh-lentivirus transduction (MOI-20, 3 days post-transduction). Fluorescence image of the eGFP of GBM14 tranduced with control-scramble-lentivirus and sh-jou-lentivirus.

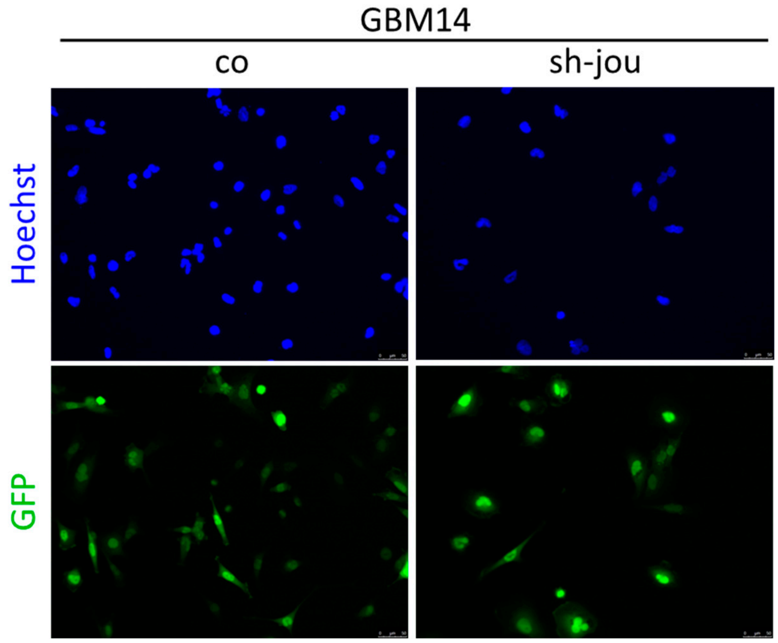

Supplementary Figure 3)

Knockdown of snoRNA-jou by sh-lentivirus reduces cells proliferation. Bright field microphotography of: A) HCT116 cells non-transduced (NT) or transduced with different MOI (MOI-20, MOI-50) after 48 hours or 72 hours. B) GBM14 cells non-transduced (NT) or transduced with different MOI (MOI-20, MOI-50) after 160 hours.

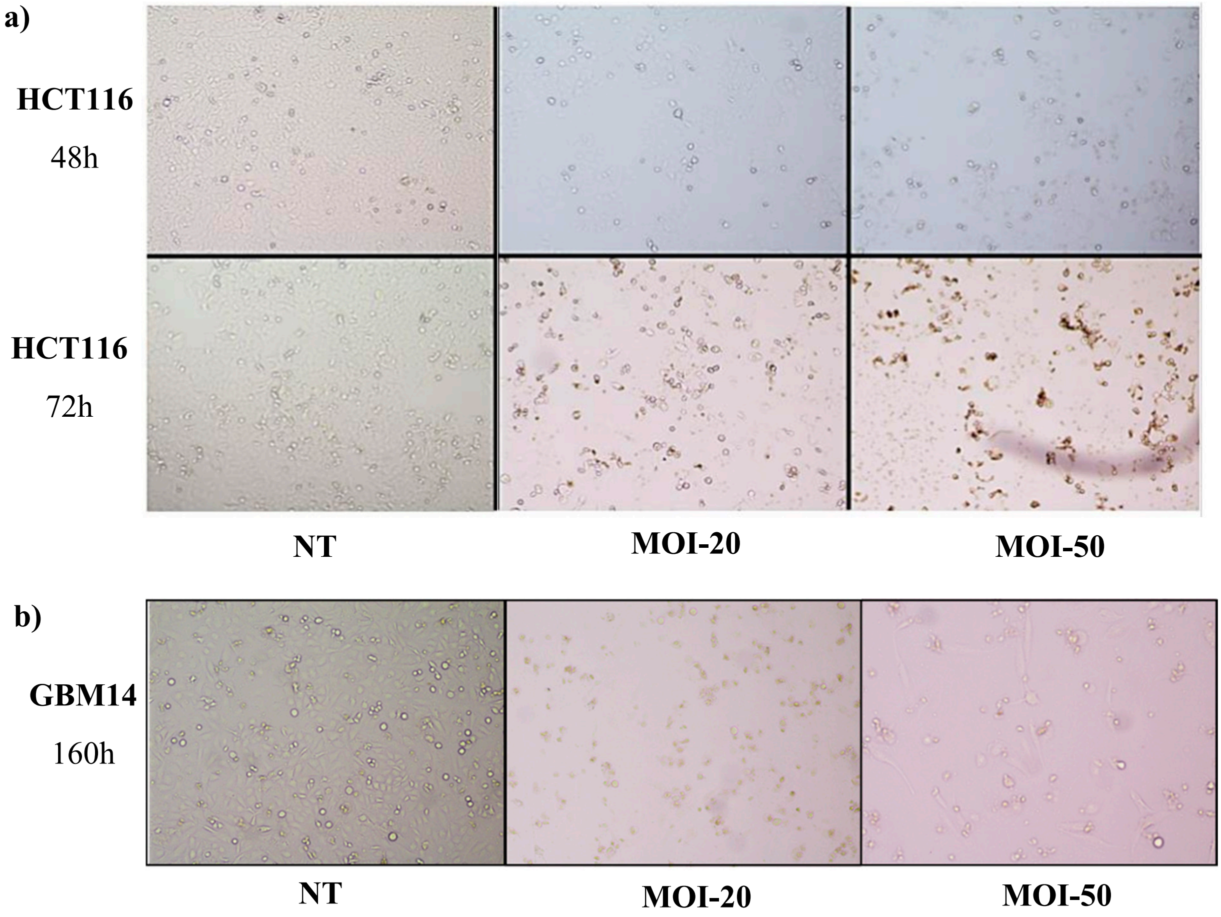

Supplementary Figure 4): Western Blots of the p21 and cyclin B1 protein expression levels following the knockdown of snoRNA-jou by sh-lentivirus on HCT-116 cells.

a) p21 and GAPDH proteins gel electrophoresis. b) Relative quantification of p21 protein expression. c) Cyclin B1 and GAPDH proteins gel electrophoresis. d) Relative quantification of cyclin B1 protein expression. Results obtained on HCT116 cells with polybrene application (Pb) and in cells transduced with sh-lentivirus (MOI-20) compared to non-transduced cells (NT). Statistics : p-values : * p < 0,05 ; *** p < 0,0005. Errors bars represent the mean +/− S.E.M. (p-value were calculated using the one-tail unpaired t-test using GraphPad Prism).

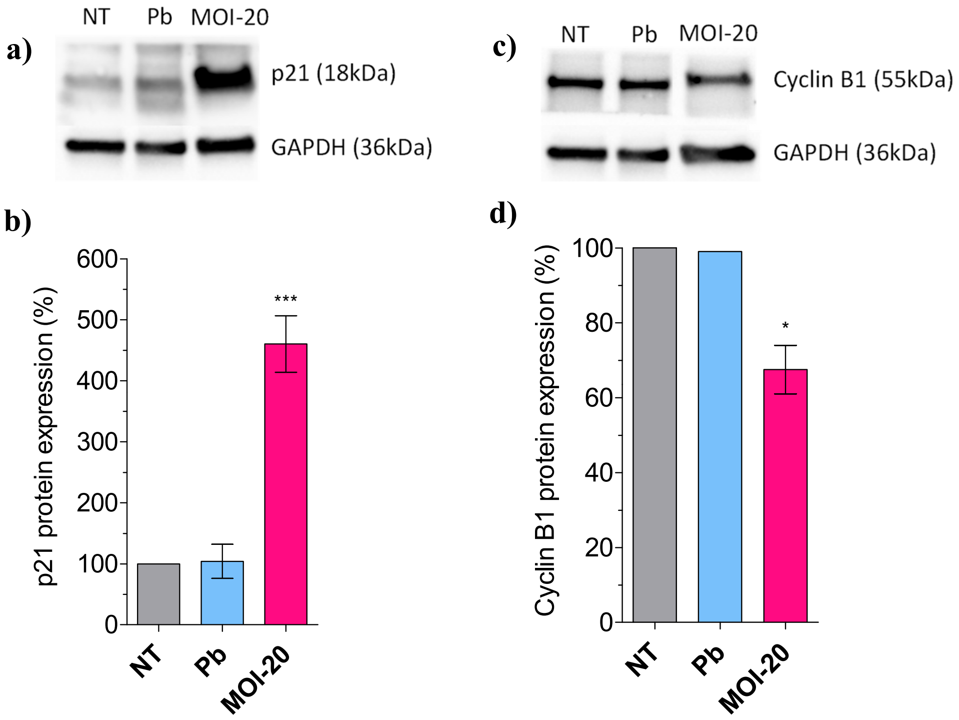

Supplementary Figure 5) Fatty-Acid (FA) composition of the different classes of Lipids and of Ester of Sterol (extracted from data of Figure 6). FA= Fatty-Acid, FA-S = FA-Saturated, FA-MU = FA-Mono-Unsaturated, FA-PU = FA-Poly-Unsaturated, FA-PU-w3 = FA-Poly-Unsaturated omega-3, FA-PU-w6 = FA-Poly-Unsaturated omega-6, w3/w6 = ratio omega-3/omega-6.

a) Fatty Acids in Total Lipids (LT)

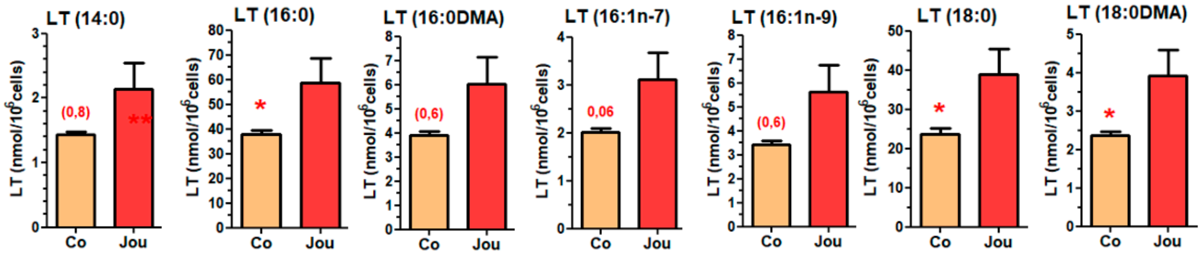

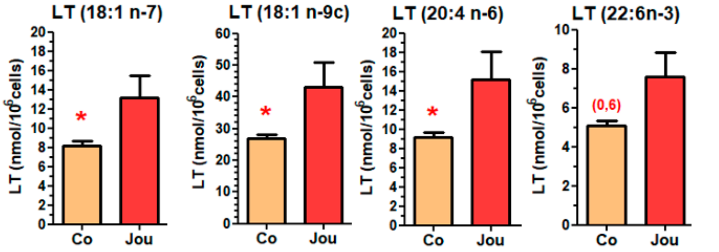

FA	Co	Jou
FA-S	69,27	109,60
FA-MU	40,36	64,97
FA-PU	9,14	15,12
FA-PU-w3	5,09	7,57
FA-PUw6	9,14	15,12
w3/w6	0,56	0,50

b) Fatty Acids in Triglycerides (TG)

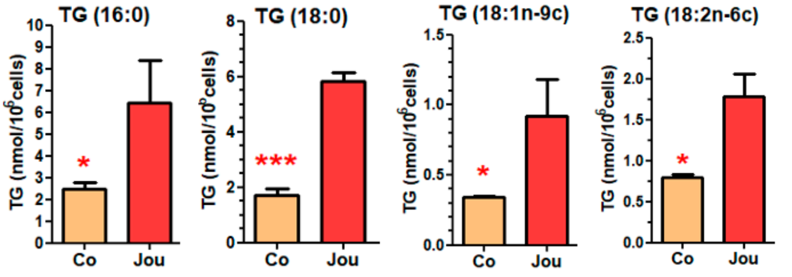

FA	Con	Jou
FA-S	4,19	10,91
FA-MU	0,34	0,92
FA-PU	0,79	1,79
FA-PUw3	0,00	0,00
FA-PUw6	0,00	0,00
w3/w6	0,00	0,00

c) Fatty Acids in Esters of Sterols (ES)

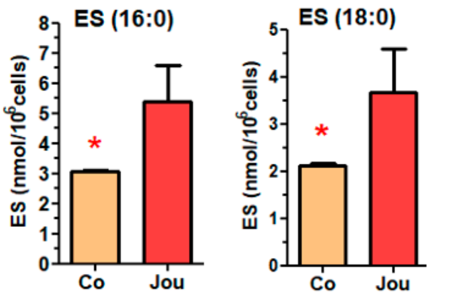

FA	Con	Jou
FA-S	5,18	6,77
FA-MU	0,35	0,00
FA-PU	0,00	0,00
FA-PUw3	0,00	0,00
FA-PUw6	0,00	0,00
w3/w6	0,00	0,00

d) Fatty Acids in Total Phospholipids (PLT)

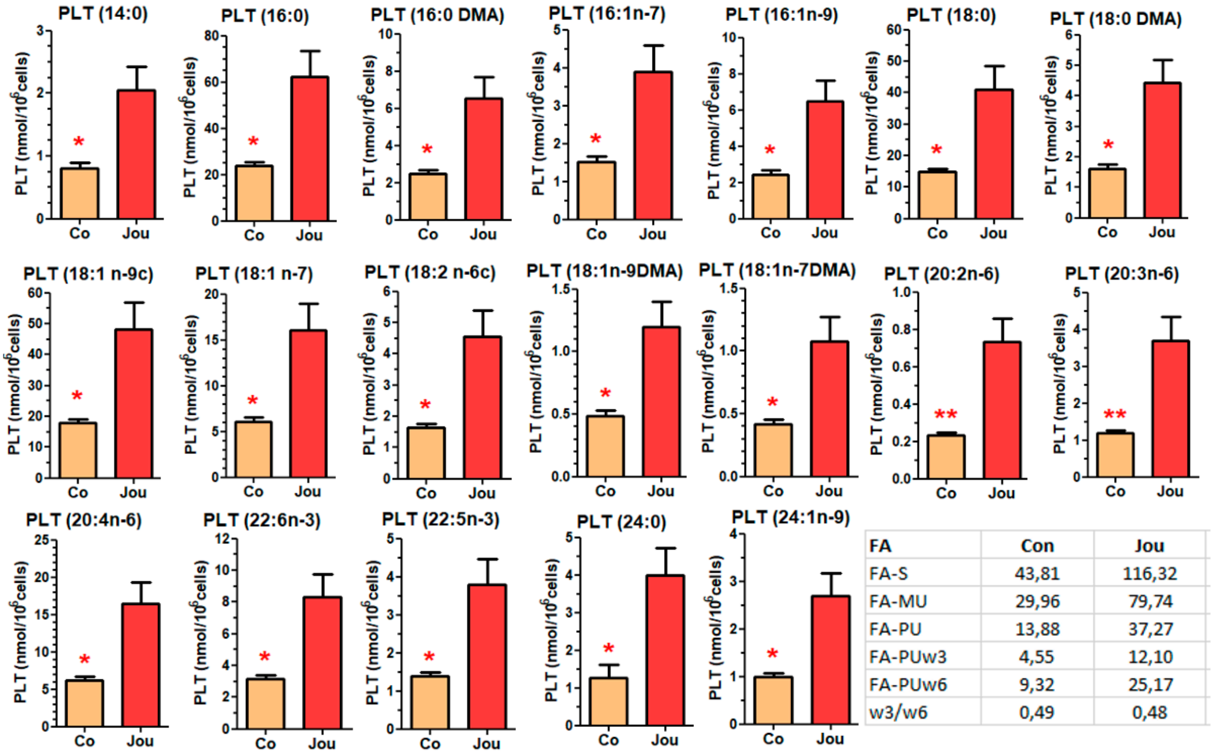

FA	Con	Jou
FA-S	43,81	116,32
FA-MU	29,96	79,74
FA-PU	13,88	37,27
FA-PUw3	4,55	12,10
FA-PUw6	9,32	25,17
w3/w6	0,49	0,48
