# Supplementary material for: Knockdown of the snoRNA-Jouvence Blocks the Proliferation and Leads to the Death of Human Primary Glioblastoma Cells
Source: Noncoding RNA. 2025 Jul 18;11(4):54. doi: 10.3390/ncrna11040054 (PMC12286020; doi:10.3390/ncrna11040054)

Supplementary Table-6

Jaque-Cabrera et al.,

CHROMATIN_SILENCING (GO_0006342)

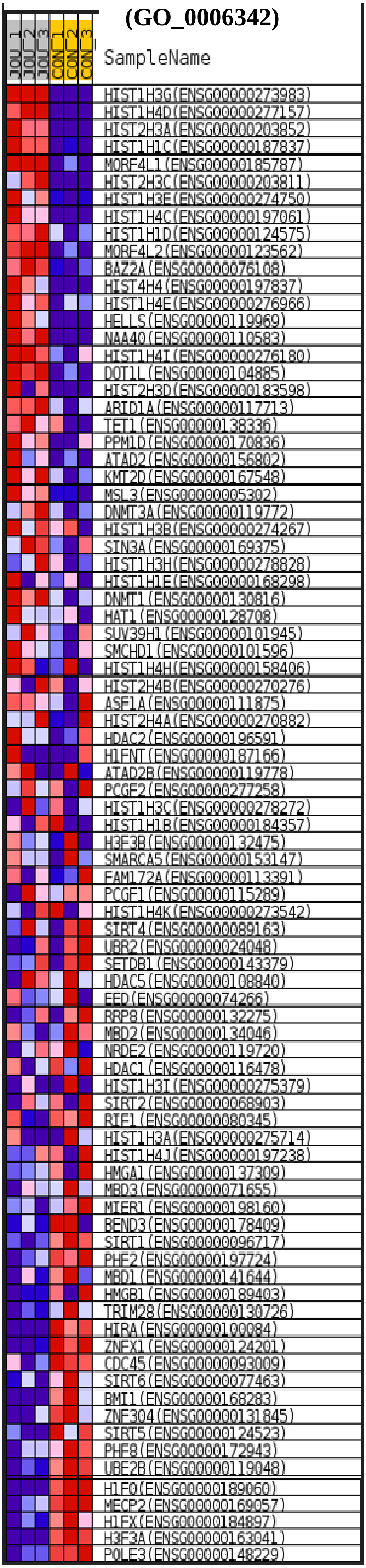

CHROMATIN_SILENCING_AT_RDNA (GO_0000183)

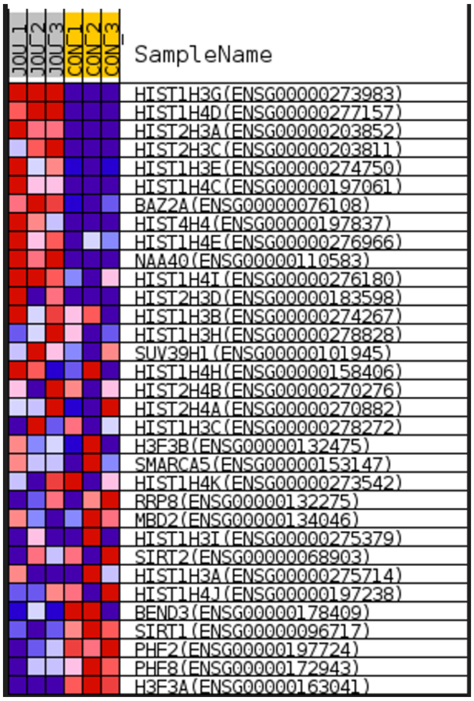

Supplement: Supplementary file 1 [file ncrna-11-00054-s001.zip › Suppl. Table-6-Chromatine Histone-Jaque Cabrera et al.pdf]
